# Whole Genome Analysis and Assessment of the Metabolic Potential of *Gordonia rubripertincta* Strain 112, a Degrader of Aromatic and Aliphatic Compounds

**DOI:** 10.3390/biology12050721

**Published:** 2023-05-15

**Authors:** Ekaterina Frantsuzova, Alexander Bogun, Viktor Solomentsev, Anna Vetrova, Rostislav Streletskii, Inna Solyanikova, Yanina Delegan

**Affiliations:** 1 Institute of Biochemistry and Physiology of Microorganisms, Federal Research Center “Pushchino Scientific Center for Biological Research of Russian Academy of Sciences” (FRC PSCBR RAS), 142290 Pushchino, Moscow Region, Russia; frantsuzova.ee@gmail.com (E.F.); bogun62@mail.ru (A.B.); solomentsev@obolensk.org (V.S.); phdvetrova@gmail.com (A.V.); innap_solyan@yahoo.com (I.S.); 2 State Research Center for Applied Microbiology and Biotechnology, 142279 Obolensk, Moscow Region, Russia; 3 Laboratory of Ecological Soil Science, Faculty of Soil Science, Lomonosov Moscow State University, 119991 Moscow, Russia; streletskiyrostislav@mail.ru; 4 Regional Microbiological Center, Belgorod State University, 308015 Belgorod, Russia

**Keywords:** *Gordonia rubripertincta*, genome assembly, biodegradation, catechol, aromatic compounds, alkanes

## Abstract

**Simple Summary:**

In the modern world, natural ecosystems are subjected to significant anthropogenic and technogenic stress. As a result of industrial processes, pollutants of various chemical structures are discharged into water and soil ecosystems. Many bacteria are able to utilize pollutants, thus contributing to the remediation of polluted ecosystems. The *Gordonia rubripertincta* strain 112 is interesting as a destroyer of aromatic and aliphatic compounds. The study of the molecular mechanisms of its functioning will allow us to use it effectively in green technologies in the future. We have shown that the strain is able to quickly cope with toxic pollutants without stress and growth inhibition.

**Abstract:**

The application of *Gordonia* strains in biotechnologies of environmental purification as degraders of pollutants of different chemical structures is an interesting research topic. The strain *Gordonia rubripertincta* 112 (IEGM112) is capable of utilizing diesel fuel, alkanes, and aromatic compounds. The aim of this work was to study the potential of *G. rubripertincta* 112 as a degrader of aromatic and aliphatic compounds and analyze its complete genome in comparison with other known *G. rubripertincta* strains. The genome had a total length of 5.28 Mb and contained 4861 genes in total, of which 4799 were coding sequences (CDS). The genome contained 62 RNA genes in total, of which 50 were tRNAs, three were ncRNAs, and nine were rRNAs. The strain bears plasmid elements with a total length of 189,570 nucleotides (plasmid p1517). The strain can utilize 10.79 ± 1.17% of hexadecane and 16.14 ± 0.16% of decane over 3 days of cultivation. In the genome of the strain, we have found metabolic pathways of alkane (cytochrome P450 hydroxylases) and catechol (ortho- and meta-pathways) degradation. These results will help us to further approach the fundamental study of the processes occurring in the strain cells and to enrich our knowledge of the catabolic capabilities of *G. rubripertincta*.

## 1. Introduction

Actinobacteria of the genus *Gordonia* are a significant part of microbial communities formed in various ecosystems, such as soils, waters, and sediments [1,2,3,4,5]. It can be said that due to their amazing metabolic flexibility, *Gordonia* strains are distributed everywhere. Representatives of the genus *Gordonia* have been isolated from the soil of the cold desert in India (*G. terrae*, EU333873.1), the sand of Namibia (*G. namibiensis*, [6]), and even from the intestinal tract of *Periplaneta americana* (*G. terrae*, [7]). Species of *Gordonia* are resistant to desiccation [8] and the metal content in the habitat [9], and they are capable of growing in saline media [10].

*Gordonia* strains are interesting for application in biotechnologies of environmental purification as degraders of pollutants of different chemical structures, such as alkanes [11,12,13,14], aromatic hydrocarbons [15,16] and their derivatives [17,18], thiophenes [19,20,21], and phthalates [22,23,24,25,26]. The type strains of several *Gordonia* species were isolated from soils contaminated with thiophenes (*G. amicalis* [27]), tar (*G. alkanivorans* [28]), from automobile tires (*G. polyisoprenivorans* [29], *G. westfalica* [30]), oil-producing wells (*G. paraffinivorans* [31]), and activated sludge [23].

Little is known about the metabolic capabilities of *Gordonia rubripertincta* representatives. The *G. rubripertincta* strain CWB2 is a promising producer of siderophores [32,33,34,35,36] and is capable of utilizing styrene [37,38,39]. A strain of the *G. rubripertincta* SD5 strain has the ability to utilize di-(2-ethylhexyl) phthalate [40]. The ability of *G. rubripertincta* to utilize alkanes has been shown only by Trögl et al. [41] who used the CWB2 strain as an example. The authors studied the microbial degradation of a C_10_-C_40_ mixture of alkanes in a model soil experiment. Thus, presently, the potential of *G. rubripertincta* strains as degraders of various pollutants has been very poorly studied.

The strain *Gordonia rubripertincta* 112 (IEGM112) was isolated in 1980 from crude oil-contaminated soil in the territory of Ukraine. The aim of this work is to study the potential of the *G. rubripertincta* strain 112 as a degrader of aromatic and aliphatic compounds and analyze its genome in comparison with other known *G. rubripertincta* strains.

The catabolism of alkanes in the representatives of the genus *Gordonia* can be proceeded by both terminal and subterminal oxidation. During terminal oxidation, alcohol, aldehyde, and acid are sequentially formed from alkanes. Thus, Liu et al. [12] observed the formation of hexadecanol and hexadecanoic acid in the cells of the *Gordonia sihwaniensis* strain utilizing hexadecane. However, the authors also recorded the presence of hexadecene in the cells, which suggested the possibility of an alternative pathway of alkane catabolism.

The subterminal oxidation of alkanes was observed by Kotani et al. [42] in *Gordonia* sp. TY-5. The strain oxidized propane to 2-propanol. Then, 2-propanol was converted to acetone, and then under the control of Baeyer–Villiger monooxygenase, it was transformed into an ester [43]. Examples of degradation of longer alkanes by *Gordonia* strains through subterminal oxidation are not currently available in the literature.

It has been reported in the literature that *Gordonia* strains use two genetic systems for terminal alkane oxidation: *alk*B for long and cytochrome P450 (CYP153) for short ones [12,13,44]. The *alk*B genetic system is usually represented by a single copy, while CYP153 can have several copies. For example, we previously showed that the *Gordonia amicalis* 1D strain contains one copy of *alk*B and two copies of CYP153, which allows it to utilize alkanes with a length range from C_10_ to C_36_ [45].

Among *Gordonia*, it is also known that there may be no *alk*B genes at all. The entire process of alkane catabolism in such strains is controlled by the alkane monooxygenases of the CYP153 family. For example, this is a characteristic of *G. alkanivorans* strains [20]. It is now known that both *alk*B and CYP153 genes are involved in alkane catabolism with a medium chain length. In actinobacteria, the *alk*B system controls the oxidation of C_16_–C_40_ alkanes, while CYP153 oxidizes alkanes with a shorter chain (shorter than C_14_) [46,47].

The ability to utilize aromatic compounds (naphthalene and its derivatives), unlike alkanes, is infrequent in *Gordonia*. Lin et al. [16] described the organization of an operon involved in naphthalene catabolism. One of the strains in our laboratory collection, *G. polyisoprenivorans* 135 [15], is also capable of utilizing naphthalene and its derivatives. The organization of the naphthalene operon in strain 135 is different from the one of *Gordonia* sp. strain CC-NAPH129-6 in the work of Lin et al. Catechol degradation among *Gordonia* was previously observed only in *G. polyisoprenivorans* [18].

In this work, we plan to study the organization of the genetic systems of alkane and catechol catabolism in the *G. rubripertincta* strain 112 and compare them with the organization of similar systems in the genomes of other *Gordonia* strains. These results will help us to further approach the fundamental study of the processes occurring in the strain cells and to enrich our knowledge of the catabolic capabilities of *G. rubripertincta*.

## 2. Materials and Methods

### 2.1. Bacterial Strain

The strain *Gordonia rubripertincta* 112 (IEGM112) can be found in the IEGM Regional Specialized Collection of Alkanotrophic Microorganisms (Perm, Russia) and in the collection of the laboratory of the physiology of microorganisms IBPM RAS (Pushchino, Moscow Region, Russia).

### 2.2. Chemicals

High-purity-grade (>98%) catechol, dichloromethane, decane, and hexadecane were obtained from Sigma-Aldrich (Burlington, MA, USA). All the other reagents were of analytical grade.

### 2.3. Growth Media and Conditions

The strain *G. rubripertincta* 112 was grown at 27 °C in a liquid mineral medium with decane (7.5 mL/L), hexadecane (7.5 mL/L), catechol (0.1 g/L), benzoate (1 g/L), or potassium acetate (10% *w*/*w*) as the sole carbon and energy source on an orbital shaker at 180 rpm.

To obtain the inoculum, the strain was grown in a mineral medium with acetate for 20 h. The biomass was precipitated and washed with sterile distilled water before inoculating it into experimental flasks. The resulting precipitate was resuspended in a mineral medium until a concentration of 10^8^ CFU/mL was reached using a turbidity standard. The inoculum was added to the experimental flasks to the final concentration of 10^6^ CFU/mL.

The following mineral medium was used in this study: K_2_HPO_4_—8.71 g/L, 5 M NH_4_Cl solution—1 mL/L, 0.1 M Na_2_SO_4_ solution—1 mL/L, 62 mM MgCl_2_ solution—1 mL/L, 1 mM CaCl_2_ solution—1 mL/L, 0.005 mM (NH_4_)_6_Mo_7_O_24_ × 4H_2_O solution, micronutrients—1 mL (micronutrient composition in g/L: ZnO—0.41 g, FeCl_3_ × 6H_2_O—5.4 g, MnCl_2_ × 4H_2_O—2 g, CuCl_2_ × 2H_2_O—0.17 g, CoCl_2_ × 6H_2_O—0.48 g, H_3_BO_3_—0.06 g), pH 7.0.

Lysogeny broth (LB) medium [48], consisting of (per liter of distilled water) 10 g of tryptone, 5 g of yeast extract, 5 g of NaCl, and 15 g of agar (PanReac, Barcelona, Spain), was used to obtain individual colonies and assess bacterial abundance.

To evaluate the efficiency of aliphatic hydrocarbon degradation by *G. rubripertincta* 112, the bacterium was cultured at 27 °C in a liquid mineral medium with decane or hexadecane for 3 days. All the experiments were performed in three independent biological replicates.

### 2.4. Determination of Hydrocarbon Concentration

The samples were analyzed using the equipment of the Collective Use Center, Soil Science Faculty, and Lomonosov Moscow State University. Decane or hexadecane was extracted from the culture medium with dichloromethane (1:2, *v*/*v*). To stop all biological processes, pre-acidification of the culture medium with sulfuric acid to pH 2 was performed. Decane and hexadecane were measured using a gas chromatography system (Agilent 6890, Agilent Technologies, Santa Clara, CA, USA) equipped with a flame ionization detector. The chromatographic column was DB-1 (30 m × 0.25 mm id, 0.25 µm). The oven temperature program was from 40 °C with an increase of 15 °C/min and 11.7 °C/min for decane and hexadecane, respectively. 

Absolute calibration with analytical standards was used for quantitation. The correlation coefficient was 0.98. The validity of the results was confirmed using one-factor analysis of variance (ANOVA), which was *p* = 0.05. The samples were diluted 100-fold before the assay was utilized. All of the results were derived from five independent replicates.

The degree of decane/hexadecane biodegradation (D) was calculated using the following formula:

D = (C_0_ − C_i_)/C_0_ × 100 [%],

where C_0_ is the concentration of hydrocarbon in the experiment without microorganisms (abiotic control), and C_i_ is the concentration of hydrocarbon in the experiment with microorganisms after 72 h of growth.

### 2.5. Genome Sequencing and Analysis

Genomic DNA was isolated from a fresh culture biomass (a colony) of *Gordonia rubripertincta* 112 grown on LB agar using a DNeasy Blood & Tissue Kit (QIAGEN, 69506). Sequencing was performed on an MGI platform (DNBSEQ-G400) using the DNBSEQ-G400RS High-throughput Sequencing Set (FCL PE150) (2 × 150 bp). A paired-end library was prepared with the MGIEasy Universal DNA Library Prep Set. We obtained 11,794,002 paired-end reads.

The raw reads were filtered using Trimmomatic v. 0.39 [49] and assembled using SPAdes v. 3.15.4 [50]. Contigs shorter than 500 bp were removed. We have obtained 87 contigs (Table 1).

The ANI value was calculated using the EzBioCloud ANI Calculator [51]. DNA-DNA hybridization (DDH) was calculated using the Genome-to-Genome Distance Calculator (GGDC) [52]. The genome was annotated with the NCBI Prokaryotic Genome Annotation Pipeline (PGAP) version 4.6 [53], Prokka [54], and RAST [55]. The genome contained 4861 genes in total, of which 4799 were coding sequences (CDS). Out of a total of 4799 CDS, 4715 were CDS with protein and 84 were without protein (pseudogenes). The genome contained 62 RNA genes in total, of which 50 were tRNAs, 3 ncRNAs, and 9 rRNAs. The genome data can be accessed in the GenBank database under the accession number JARUXG000000000 (BioProject PRJNA953757, BioSample SAMN34123077).

The whole-genome tree was built using the TYGS web service [https://tygs.dsmz.de/ (accessed on 1 May 2023)]. TYGS uses FastME 2.1.4 [56] to build the trees from Genome BLAST Distance Phylogeny (GBDP) distances calculated from genome sequences [57] and the “greedy-with-trimming” algorithm [58]. Alignment maps were built using Mauve ver. 2.4.0, (21 December 2014) [59]. For pangenome analysis and unique genes search, we used OrthoVenn [60]. The functional annotation of the genome was carried out using KEGG [61]. The search for clusters of secondary metabolite production was carried out using Antismash [62]. CheckM v. 1.2.2 [63] was used for assessing the quality of genomes. Metabolic pathways were drawn using ChemDraw Ultra ver. 12.0.2.1076.

## 3. Results and Discussion

### 3.1. Identification of Strain 112

On agarized media, strain 112 forms small round colonies of a pink–orange color (Figure S1). The strain can grow within a temperature range from 10 to 40 °C (optimum 28–30 °C) and within a pH range from 6.3 to 8.4 (optimum 7.3). Strain 112 was originally assigned to the genus *Rhodococcus* [64], but it was later reidentified as presumably belonging to the species *Gordonia rubripertincta*. For a more reliable identification, we calculated the ANI and DDH parameters between the genomes of strain 112 and the *G. rubripertincta* strain ATCC14352. In addition, we compared the genome of strain 112 with other *G. rubripertincta* strains from the Genbank database.

As of now (April 2023), there are eight *G. rubripertincta* genomes in the Genbank database, including strain 112 (https://www.ncbi.nlm.nih.gov/genome/browse#!/prokaryotes/12310/ (accessed on 30 April 2023)) (Table 2).

The sequences which were designated to be ATCC14352 (JAAXPB000000000.1) and NBRC101908 (BAHB00000000.1) belong to the same strain, the type strain of *G. rubripertincta*. For a further work, we took the ATCC14352 (JAAXPB000000000.1) genome (Table 3). The genome of strain IEGM1388 (JAPWIE000000000.1) has a very low DDH value with the genome of the *G. rubripertincta* strain ATCC14352 (14.60%), which led us to assume that strain IEGM1388 does not belong to *G. rubripertincta* at all. A BLAST search for 16S rRNA and *gyr*B gene sequences showed that strain IEGM1388 is a member of the genus *Williamsia*, its closest relative being *Williamsia* sp. NRRL B-15444R (JN201861.1). We do not consider strain IEGM1388 to be *G. rubripertincta* hereafter.

**Figure 1:**
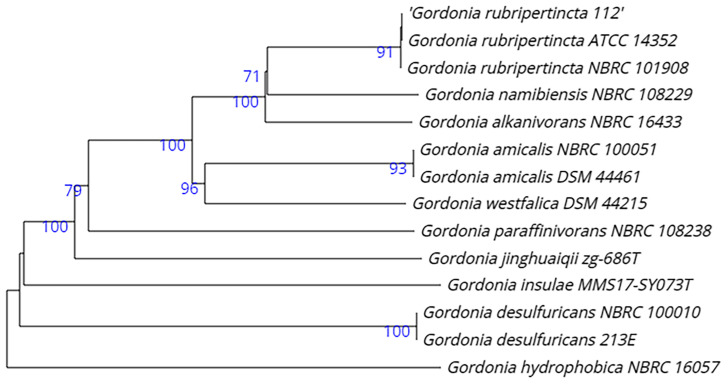
Whole-genome tree demonstrating the position of strain 112 within the genus *G. rubripertincta* and within the genus *Gordonia* in general.

On the phylogenetic tree, strain 112 is also clustered with *G. rubripertincta* (Figure 1).

Thus, strain 112 reliably belongs to *G. rubripertincta*. Its closest relative is the *Gordonia rubripertincta* type strain ATCC14352.

### 3.2. The Plasmid of the Gordonia rubripertincta Strain 112

**Figure 2:**
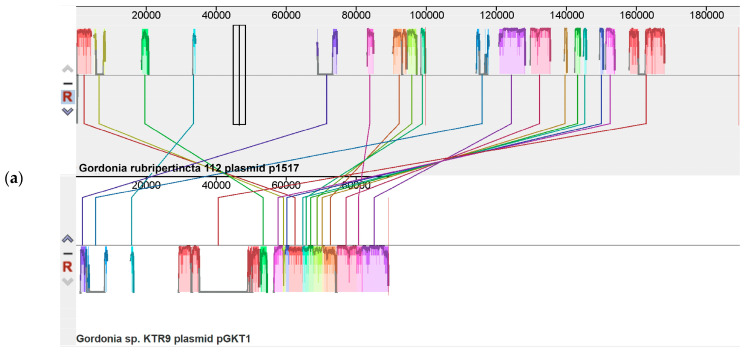

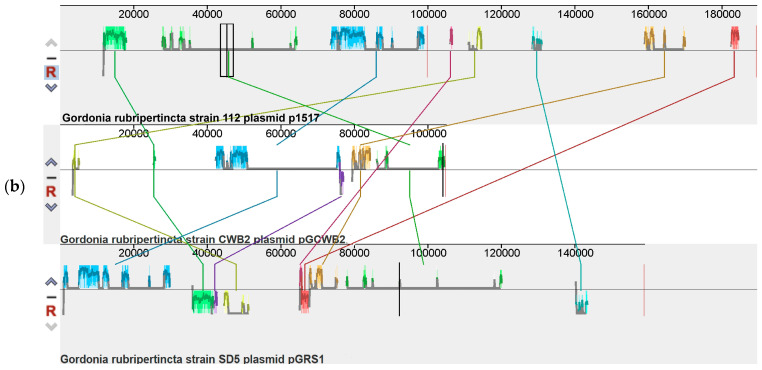
Mauve visualization of locally collinear blocks identified between p1517 of *G. rubripertincta* 112 and relative plasmids: (**a**) pGKT1 of *Gordonia* sp. KTR9, (**b**) pGCWB2 *of G. rubripertincta* CWB2 and pGRS1 of *G. rubripertincta* SD5. Vertical bars mark boundaries between elements.

Contigs 15 and 17 of strain 112 were identified as plasmid elements with a total length of 189,570 nucleotides (plasmid p1517). Plasmid p1517 is most related to plasmid pGKT1 of *Gordonia* sp. KTR9 (NC_018582.1) (Figure 2a). No significant kinship was observed with the plasmids of *G. rubripertincta* SD5 and CWB2 strains (Figure 2b).

Plasmid p1517 of the *G. rubripertincta* strain 112 is predominantly composed of genes encoding hypothetical proteins. It also contains genes encoding mobile genome elements, plasmid maintenance, and separation genes. The genes responsible for metal transport (lead, cadmium, zinc, mercury, manganese) and metal resistance (cadmium, cobalt, zinc) are localized on plasmid p1517.

Plasmids pGCWB2 of *G. rubripertincta* CWB2 and pGRS1 of *G. rubripertincta* SD5, unlike plasmid p1517 of strain *G. rubripertincta* 112, contain catabolic genes. Plasmid pGCWB2 (CP022581.1) contains:

1. Phenylacetate catabolism gene cluster;
2. Styrene monooxygenase *Sty*A, 3 beta-hydroxysteroid dehydrogenase/Delta 5-->4-isomerase;
3. Genes involved in type IV secretory system Conjugative DNA transfer.

Plasmid pGRS1 of *G. rubripertincta* SD5 contains genes of the aromatic compound catabolism pathway, namely aromatic ring-hydroxylating dioxygenase (alpha and beta subunits), alpha/beta fold hydrolase, extradiol dioxygenase, and ferredoxin.

### 3.3. Pangenome Analysis of Gordonia rubripertincta Strains

The pangenome of strain 112 and its closest relatives (DDH > 80%) *G. rubripertincta* ATCC14352, CWB2, and W3S5 was analyzed. All genomes used for pangenome analysis had completeness above 99% and contamination levels of 0.03–0.54%.

**Figure 3:**
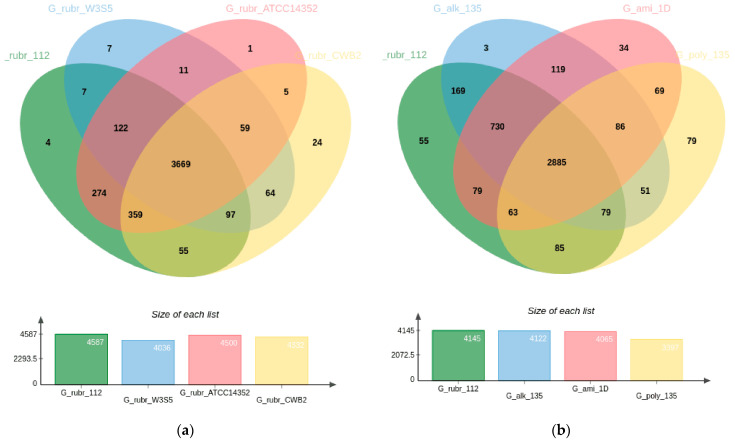
Venn diagram depicting pangenome of (**a**) *Gordonia rubripertincta* strains 112, ATCC14352, CWB2 and W3S5, (**b**) *G. rubripertincta* 112, *G. amicalis* 1D, *G. alkanivorans* 135, and *G. polyisoprenivorans* 135.

The pangenome of the strains is represented by 4758 genes, of which 3669 of the genes (77.11%) are core (genes that all our strains have). The result obtained may indicate the heterogeneity of *G. rubripertincta* species (Figure 3). For comparison, in a previous study [65], we showed that *R. qingshengii* accounts for 86% of the pangenome.

The CWB2 strain has the greatest number of unique coding sequences among the four *G. rubripertincta* strains analyzed. Among the 24 amino acid sequences unique to it and not found in three other strains (Figure 3a), there are products with nitrilotriacetate monooxygenase activity (four amino acid sequences), oxidoreductase activity (five aa sequences), and aflatoxin biosynthetic process (two aa sequences). Strain 112 has four unique amino acid sequences, but their function could not be determined. Strain 112 and ATCC14352 are the closest relatives of all *G. rubripertincta* studied, so we expected to see the greatest number of amino acid sequences unique to the pair (274 sequences, Table S1A).

In addition, we studied the pangenome of *Gordonia* strains from other species in our laboratory collection. All the strains taken for analysis had one thing in common: the ability to degrade alkanes. Strain *G. polyisoprenivorans* 135 utilized chloroaromatic compounds [18], strain *G. alkanivorans* 135 utilized thiophenes as the sole source of sulfur [20], and strain *G. amicalis* 1D utilized alkanes up to C_36_ [45]. Strain *G. rubripertincta* 112 was the only one of all the listed strains that utilized short alkanes (C_8_-C_12_) more actively than alkanes with a C_16_+ chain length.

The pangenome of the strains of four different *Gordonia* species is 4586 genes, of which 2885 (62.90%) derive from the core (Figure 3b). Among the different *Gordonia* species analyzed, strain *G. rubripertincta* 112 is the most related to strain *G. alkanivorans* 135. The G_rubr_112/G_alk_135 pair has 169 genes unique to the pair which were not found in the other strains analyzed (Table S1B). Among the genes unique to the G_rubr_112/G_alk_135 pair, it is interesting to note the genes belonging to the following categories (Table 4).

A BLAST search of the nucleotide (and amino acid, with similar results) sequence of the 01466 gene of strain *G. rubripertincta* 112 showed that this sequence with the parameters query cover > 90% and per. ident > 90% is characteristic only of some (not all) of the strains of *G. rubripertincta* and *G. alkanivorans* (Table 5).

Changing the search mode from highly similar sequences (megablast) to somewhat similar sequences (blastn) reveals organisms with sequences remotely related to the gene, but their query coverage and percent identity are too low (<70%) to speak of unconditional relatedness. The product of chorismatase catalyzes the hydrolysis of isochorismate into 2,3-dihydro-2,3-dihydroxybenzoate and pyruvate [66].

The steroid catabolism genes (GO:0006694, GO:0008202, GO:0006707) in both strains are located one after another and presumably represent an operon of the following structure: flavin-dependent monooxygenase, reductase subunit HsaB → 4,5:9,10-diseco-3-hydroxy-5,9,17-trioxoandrosta-1(10),2-diene-4-oate hydrolase → hypothetical protein → 3-ketosteroid-9-alpha-monooxygenase, ferredoxin reductase component → 3-oxosteroid 1-dehydrogenase. A BLAST search for the gene sequences of this operon showed that they, similarly to isochorismatase, occur in individual representatives of *G. rubripertincta* and *G. alkanivorans*.

There is no information in the literature on the phenotypic traits or the ability of *G. alkanivorans* to utilize steroids. However, the genes responsible for this ability are found in them. Besides *G. alkanivorans*, the gene cluster *kst*, responsible for the catabolism of steroid compounds, is found in the following representatives of the genus *Gordonia*: *G. rubripertincta*, *G. bronchialis*, *G. insulae*, *G. mangrovi*, *G. araii*, *G. namibiensis*, *G. sputi*, *G. crocea*, and others. An experimental confirmation of this ability in *Gordonia* was performed for *G. cholesterolivorans* [67] and *Gordonia neofelifaecis* [68,69]. The process of the catabolism of steroid compounds has been studied in the most detail in the strain *G. rubripertincta* CWB2 strain [38]. Our strain *G. rubripertincta* 112, similar to other representatives of this species, possesses a set of genes for steroid catabolism; however, the study of the phenotypic manifestations of this process in strain 112 was not included in the task of this work.

### 3.4. Functional Annotation of the Genome of the Strain 112

**Figure 4:**
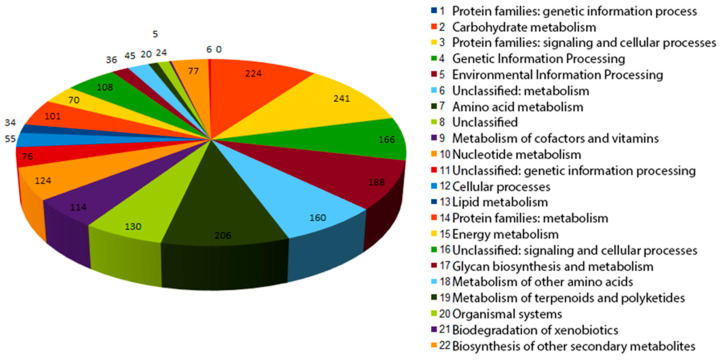
Number of genes associated with general functional categories in the genome of strain 112 based on KEGG classification.

Out of 4787 genes, 2212 (46.2%) were functionally annotated (Figure 4).

The result of the functional annotation shows that strain 112 possesses the genes of all metabolic pathways required for autonomous culture existence. We also performed functional annotation of the genomes of other *G. rubripertincta* from the Genbank database (Table 6) to compare the representation of the metabolic pathways of interest in the genomes of the strains of this species.

The «Xenobiotics degradation and metabolism» category in strain 112 contains 77 genes. The most frequently occurring genes (>10 genes) in the category are the following functional clusters:

Benzoate degradation—23 CDS.

Xylene degradation—10 CDS.

Steroid degradation—11 CDS.

#### 3.4.1. Diversity of Aromatic Compound Catabolism Genes in the Genome of *Gordonia rubripertincta* 112

We observed in strain 112 the ability to grow on benzoate and catechol as the sole source of carbon and energy. Catechol degradation by the strain can occur via both the ortho- and meta-pathways; the corresponding genes are found in the strain genome (Figure S2).

The xylene metabolism category contains no genes encoding the enzymes of the first reaction of this process. In addition, we did not find the strain’s ability to grow on xylenes. The metabolic pathway for xylene degradation in strain 112 begins from the middle, with the methylbenzoate conversion reaction catalyzed by benzoate/toluate 1,2-dioxygenase [EC:1.14.12.10 1.14.12.-]. One of the main metabolites of this process is methylcatechol (three- or four-substituted), and the further pathway is similar to catechol catabolism.

The genomes of all *G. rubripertincta* strains contain two copies of the catechol 1,2-dioxygenase (C1,2DO) genes (Table 7). The copies have 86% query coverage and 58.33% identity between them.

Genes of the catechol catabolism meta-pathway (in particular, catechol 2,3-dioxygenase (C2,3DO)) are present in three of the six *G. rubripertincta* strains. The amino acid sequence of the C2,3DO gene in strain W3S5 differs from that in strains ATCC14352 and 112. At 99% coverage, the percent identity between them is 83.02%.

There are currently 55 known species of the genus *Gordonia* (https://lpsn.dsmz.de/ (accessed on 30 March 2023)). Genes of only the ortho-pathway of catechol catabolism (in particular, C1,2DO) are present in the genomes of the strains of 22 species (Table S2). Genes of both ortho (C1,2DO) and meta (C2,3DO) pathways were found in the genomes of the representatives of 10 species. The prevalence of C1,2DO in *Gordonia* genomes allows this gene to be used as a phylogenetic marker for the identification of strains of this genus. Shen et al. [70] suggested that the *cat*A gene encoding C1,2DO evolves faster than the rrn operon or *gyr*B gene, so *cat*A is a more sensitive marker for species identification. *Gordonia malaquae* is the only species in the genus *Gordonia* whose strains have only C2,3DO genes but no C1,2DO genes.

The enzymatic activity of C2,3DO in the work of Silva et al. [17] in the *G. polyisoprenivorans* strain was higher than that of C1,2DO under most of the conditions tested (pH, temperature, time course, ion effect). However, it is worth noting that the authors used complex LB medium with anthracene supplementation to cultivate the strain. Considering that LB medium components are more available, and a preferable substrate compared to anthracene, it is impossible to draw unequivocal conclusions about the process of aromatic compound utilization and enzyme activity under such conditions. Solyanikova et al. [71] also showed that when the cells of the *G. polyisoprenivorans* strain grew on a medium with benzoate, the activity of catechol 1,2-dioxygenase was 0.850 U/(mg of protein), and the activity of catechol 2,3-dioxygenase was absent. Thus, the simultaneous maintenance of the two metabolic pathways of catechol catabolism in *Gordonia* genomes does not mean that both pathways will be involved. Moreover, C2,3DO may be a redundant metabolic pathway in *Gordonia* strains.

**Figure 5:**
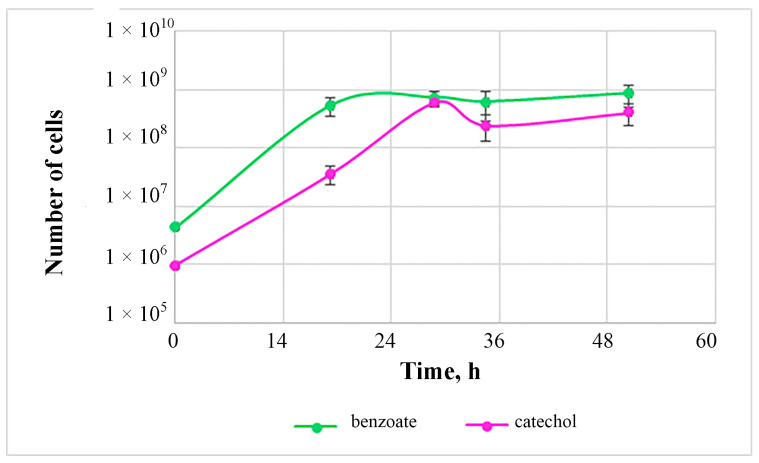
Growth curve of strain 112 on mineral medium with benzoate (green) and catechol (purple).

The cell number during growth on catechol reaches a maximum after 24 h of growth, after which the culture enters the stationary phase (Figure 5).

When growing on benzoate, we observed the maximum number of cells after 30 h of growth. Thus, when utilizing aromatic compounds, the development of the periodic culture of strain 112 proceeds rather quickly, which allows us to consider the biotechnological promise of this strain for the utilization of these compounds.

#### 3.4.2. Diversity of Alkane Catabolism Genes in Strain *Gordonia rubripertincta* 112

Strain 112 is capable of utilizing alkanes with a C_10_-C_16_ chain length, and the growth on short alkanes (decane (C_10_) as an example) is observed not only in vapors, but also in direct contact of the microorganism with the substrate. Growth on alkanes longer than C_16_ was not observed in the strain; therefore, we assumed that it, similarly to *G. alkanivorans* strains, lacked *alk*B genes, and its ability to degrade alkanes was controlled by CYP153 genes. An analysis of the annotated genome confirmed our assumptions: strain 112 indeed lacks *alk*B genes.

We analyzed the genomes of the other *G. rubripertincta* strains from the Genbank database to understand whether the absence of *alk*B genes is a strain feature or a species-specific pattern. All *G. rubripertincta* strains whose genomes were sequenced lack the *alk*B operon.

The genome of strain 112 revealed four operons with a structure typical of the alkane hydroxylating cluster of actinobacteria: cytochrome P450 hydroxylase, ferredoxin, and ferredoxin reductase. An analysis of the prevalence of hydroxylase genes from these operons among *Gordonia* showed that one of them (PRJNA953757:QBL07_23005) is not found in other *Gordonia*. The presence of this gene is characteristic of some *Rhodococcus* (Table 8), so we can assume that this operon CYP153 was obtained by strain 112 as a result of a horizontal transfer from *Rhodococcus* strains.

The GC content of the region where the described operon is located (NODE_27_length_46626) is 65.7%, which is 2% lower than the GC content of the contigs that have the highest affinity to the genome regions of the typical strain *G. rubripertincta*. This observation suggests that some elements of contig 27 were acquired during horizontal transfer.

### 3.5. Peculiarities of Alkane Catabolism by Strain G. rubripertincta 112

It is known that the toxicity of the aliphatic hydrocarbons decreases with an increasing number of carbon atoms due to a decrease in their volatility [72]. Over 3 days, the abiotic loss of decane and hexadecane in the system without microorganisms was 62.87% and 1.21%, respectively (Table 9).

The degree of degradation of decane and hexadecane relative to the control system without microorganisms was 10.79 and 16.14%, respectively. It can be noted that, despite the high abiotic loss of decane, the degree of its degradation was higher compared to the degree of hexadecane degradation.

Considering that the genome of strain 112 lacks *alk*B genes, and only cytochrome P450 hydroxylases are used for alkane degradation, it was not surprising that the strain is unable to oxidize alkanes longer than C_16_. Its abilities as an alkane degrader could be exploited in soils contaminated with light fractions of oil and petroleum products. The alkane catabolism genes in strain 112 are located on the chromosome, which suggests their stable maintenance during both non-selective cultivation and remediation processes in soil. In the genomes of other known *G. rubripertincta* strains, alkane destruction genes are also localized on chromosomes; however, there are no data on the ability of these strains to utilize alkanes. Thus, at this time, only *G. rubripertincta* 112 and CWB2 have experimentally confirmed the ability to utilize alkanes. As for catechol degradation, strain 112 is currently the only representative of *G. rubripertincta* in which this ability has been experimentally confirmed.

## 4. Conclusions

This study provides an improved understanding of the genomic organization of the *Gordonia rubripertincta* strain and its metabolic capabilities. The ability of the strain of this genus to utilize aromatic compounds was confirmed experimentally for the first time, and the genetic pathways involved in this process were described. The ability to utilize alkanes of different chain lengths, including short ones (decane), was described. The results provide a powerful basis for further transcriptome experiments and the study of the regulation of the catabolism of various pollutants by *G. rubripertincta* 112.

## Figures and Tables

**Table 1:** Metrics of the genome assembly of the strain *G. rubripertincta* 112.

Parameter	Length
Genome size, bp	5,281,129
The longest contig, bp	845,133
The shortest contig, bp	502
N50, bp	233,583
N75, bp	130,856
N90, bp	59,209

**Table 2:** Comparison of *G. rubripertincta* genomes deposited in the Genbank database.

GenBank Accession Number	Strain Name	Chromosome Size, Mb	Plasmid Size, kb
JARUXG000000000	*Gordonia rubripertincta* 112	5.09	189
JAAXPB000000000.1	*Gordonia rubripertincta* ATCC14352T	5.70	-
JAFFGU000000000.1	*Gordonia rubripertincta* BP-295	5.15	-
CP022580.1	*Gordonia rubripertincta* CWB2	5.23	105

**Table 3:** ANI and DDH values between *Gordonia rubripertincta* 112 and relative strains.

Strain Name	ANI Value, %	DDH Value, %
*Gordonia rubripertincta* ATCC14352T	99.98	98.90
*Gordonia rubripertincta* BP-295	98.18	78.40
*Gordonia rubripertincta* CWB2	98.08	86.40
*Gordonia rubripertincta* SD5	98.43	77.70
*Gordonia rubripertincta* W3S5	98.06	81.80

**Table 4:** Genes unique for the pair of strains G_rubr_112/G_alk_135 and their functions.

Gene Onthology (GO) Category Identifier	Function	Gene Identifier According to the GenBank Annotation	
		112	135
0019439	aromatic compound catabolic process	01466	00188
0006707	cholesterol catabolic process	00126	03312
0008202	steroid metabolic process	00127	03311
0006694	steroid biosynthetic process	00124 00123	03314 03315
0009712	catechol-containing compound metabolic process	01163	01469

**Table 5:** Frequency of 01466 isochorismatase gene in *Gordonia*. The sequence from strain 112 was taken as a reference.

Strain	Sequence Name	Query Cover, %	Per. Ident, %
*G. alkanivorans* 135	isochorismatase family protein	100	100
*G. rubripertincta* SD5	isochorismatase family protein	100	97.29
*G. alkanivorans* YC-RL2	isochorismatase	100	96.68
*G. alkanivorans* GH-1	isochorismatase	100	96.68
*G. rubripertincta* CWB2	maleamate amidohydrolase	69	96.98

**Table 6:** Number of functionally annotated genes in genomes of known *G. rubripertincta* strains. In cases where the strain genome is assembled to the complete level and has plasmids, the data are presented in the sum for the chromosome and plasmid.

GenBank Accession Number	Strain Name	Total Number of Genes	Functionally Annotated Genes	Functionally Annotated Genes, %
JARUXG000000000	*Gordonia rubripertincta* 112	4787	2212	46.2
JAAXPB000000000.1	*Gordonia rubripertincta* ATCC14352T	5023	2352	46.8
JAFFGU000000000.1	*Gordonia rubripertincta* BP-295	4650	2086	44.9
CP022580.1	*Gordonia rubripertincta* CWB2	4707	2092	44.4
CP059694.1	*Gordonia rubripertincta* SD5	4670	2058	44.1
VLNS00000000.1	*Gordonia rubripertincta* W3S5	4252	1993	46.9

**Table 7:** Representation of catechol dioxygenase genes in genomes of *G. rubripertincta* strains.

Strain	C1,2DO	C2,3DO
ATCC14352	2	1
W3S5	2	1
BP-295	2	-
SD5	2	-
CWB2	2	-
112	2	1

**Table 8:** Strains in whose genomes the genes most related to the CYP153 hydroxylase gene PRJNA953757:QBL07_23005 were found.

Strain	Genbank Acc Number	Query Cover, %	Percent Identity, %
*Rhodococcus pseudokoreensis* R79	CP070619	93	74.96
*Rhodococcus* sp. USK10	CP076048	94	74.80
*Rhodococcus opacus* B4	AP011115	94	74.19

**Table 9:** Abiotic loss and degree of degradation of aliphatic hydrocarbons by *G. rubripertincta* strain 112.

Growth Substrate	Abiotic Loss, %	Degradation Degree, %
hexadecane	1.21 ± 0.13	10.79 ± 1.17
decane	62.87 ± 0.41	16.14 ± 0.16

## Data Availability

The data on genome sequence of the strain *Gordonia rubripertincta* 112 can be found in the Genbank database under the accession number JARUXG000000000 (BioProject PRJNA953757, BioSample SAMN34123077).

## Supplementary Materials

The following supporting information can be downloaded at: https://www.mdpi.com/article/10.3390/biology12050721/s1, Figure S1: Growth of *G. rubripertincta* strain 112 (a) on agarized LB medium, (b) in liquid mineral medium with decane (left flask) and hexadecane (right flask); Table S1A: Biological process summary of genes unique for pair G_rubr_112/G_rubr_ATCC14352, Table S1B: Biological process summary of genes unique for pair G_rubr_112/G_alk_135; Figure S2: Schemes of (a) ortho- and (b) meta-pathways of catechol degradation; Table S2: Distribution of catechol dioxygenase genes in the genomes of representatives of different *Gordonia* species.
