# Supplementary material for: Whole Genome Analysis and Assessment of the Metabolic Potential of Gordonia rubripertincta Strain 112, a Degrader of Aromatic and Aliphatic Compounds
Source: Biology (Basel). 2023 May 15;12(5):721. doi: 10.3390/biology12050721 (PMC10215345; doi:10.3390/biology12050721)
Supplement: Supplementary file 1 [file biology-12-00721-s001.zip › biology-2380773-supplementary.pdf]

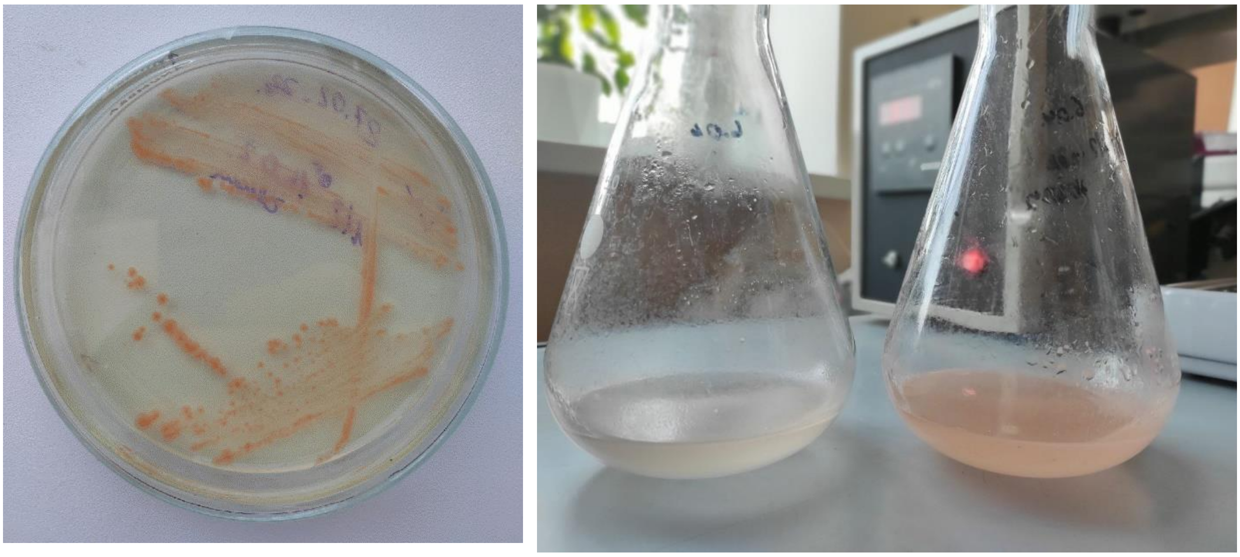

(a) (b)

Figure S1. Growth of *G. rubripertincta* strain 112 (a) on agarized LB medium, (b) in liquid mineral medium with decane (left flask) and hexadecane (right flask).

Table S1A. Biological process summary of genes unique for pair G_rubr_112/G_rubr_ATCC14352

GO Category Number	Function	Number of Genes
GO:0008152	metabolic process	39
GO:0008150	biological_process	35
GO:0044237	cellular metabolic process	26
GO:0006629	lipid metabolic process	13
GO:0006725	cellular aromatic compound metabolic process	12
GO:0006807	nitrogen compound metabolic process	12
GO:0043170	macromolecule metabolic process	11
GO:0044238	primary metabolic process	11
GO:0009987	cellular process	10
GO:0006082	organic acid metabolic process	9
GO:0046483	heterocycle metabolic process	9
GO:0050896	response to stimulus	8
GO:0006139	nucleobase-containing compound metabolic process	7
GO:0065007	biological regulation	6
GO:0016070	RNA metabolic process	5
GO:0006811	ion transport	4
GO:0006259	DNA metabolic process	3
GO:0016043	cellular component organization	3
GO:0044255	cellular lipid metabolic process	3
GO:0006805	xenobiotic metabolic process	2
GO:0006865	amino acid transport	2
GO:0019538	protein metabolic process	2
GO:0032502	developmental process	2
GO:0034622	cellular macromolecular complex assembly	2
GO:0045333	cellular respiration	2
GO:0065003	macromolecular complex assembly	2
GO:0005975	carbohydrate metabolic process	1
GO:0006066	alcohol metabolic process	1
GO:0006119	oxidative phosphorylation	1
GO:0006281	DNA repair	1
GO:0006304	DNA modification	1
GO:0006396	RNA processing	1
GO:0006508	proteolysis	1
GO:0006793	phosphorus metabolic process	1
GO:0006810	transport	1
GO:0007005	mitochondrion organization	1
GO:0007154	cell communication	1
GO:0015849	organic acid transport	1
GO:0017144	drug metabolic process	1
GO:0032196	transposition	1
GO:0032989	cellular component morphogenesis	1
GO:0051186	cofactor metabolic process	1
GO:0051189	prosthetic group metabolic process	1
GO:0051234	establishment of localization	1
GO:0051704	multi-organism process	1

Table S1B. Biological process summary of genes unique for pair G_rubr_112/G_alk_135

GO Category Number	Function	Number of Genes
GO:0008152	metabolic process	32
GO:0008150	biological_process	29
GO:0044237	cellular metabolic process	21
GO:0006807	nitrogen compound metabolic process	13
GO:0006629	lipid metabolic process	11
GO:0006725	cellular aromatic compound metabolic process	11
GO:0044238	primary metabolic process	11
GO:0006139	nucleobase-containing compound metabolic process	9
GO:0043170	macromolecule metabolic process	9
GO:0046483	heterocycle metabolic process	9
GO:0006082	organic acid metabolic process	8
GO:0009987	cellular process	6
GO:0006810	transport	5
GO:0016070	RNA metabolic process	5
GO:0051234	establishment of localization	5
GO:0065007	biological regulation	5
GO:0006259	DNA metabolic process	4
GO:0006811	ion transport	4
GO:0032196	transposition	4
GO:0044255	cellular lipid metabolic process	4
GO:0006066	alcohol metabolic process	2
GO:0006518	peptide metabolic process	2
GO:0015074	DNA integration	2
GO:0043603	cellular amide metabolic process	2
GO:0006281	DNA repair	1
GO:0006412	translation	1
GO:0006793	phosphorus metabolic process	1
GO:0006865	amino acid transport	1
GO:0008643	carbohydrate transport	1
GO:0009116	nucleoside metabolic process	1
GO:0009117	nucleotide metabolic process	1
GO:0009308	amine metabolic process	1
GO:0015849	organic acid transport	1
GO:0016043	cellular component organization	1
GO:0017144	drug metabolic process	1
GO:0019538	protein metabolic process	1
GO:0032502	developmental process	1
GO:0032989	cellular component morphogenesis	1
GO:0042180	cellular ketone metabolic process	1
GO:0045229	external encapsulating structure organization	1
GO:0050896	response to stimulus	1
GO:0051179	localization	1
GO:0051186	cofactor metabolic process	1
GO:0051704	multi-organism process	1

(a)

(b)

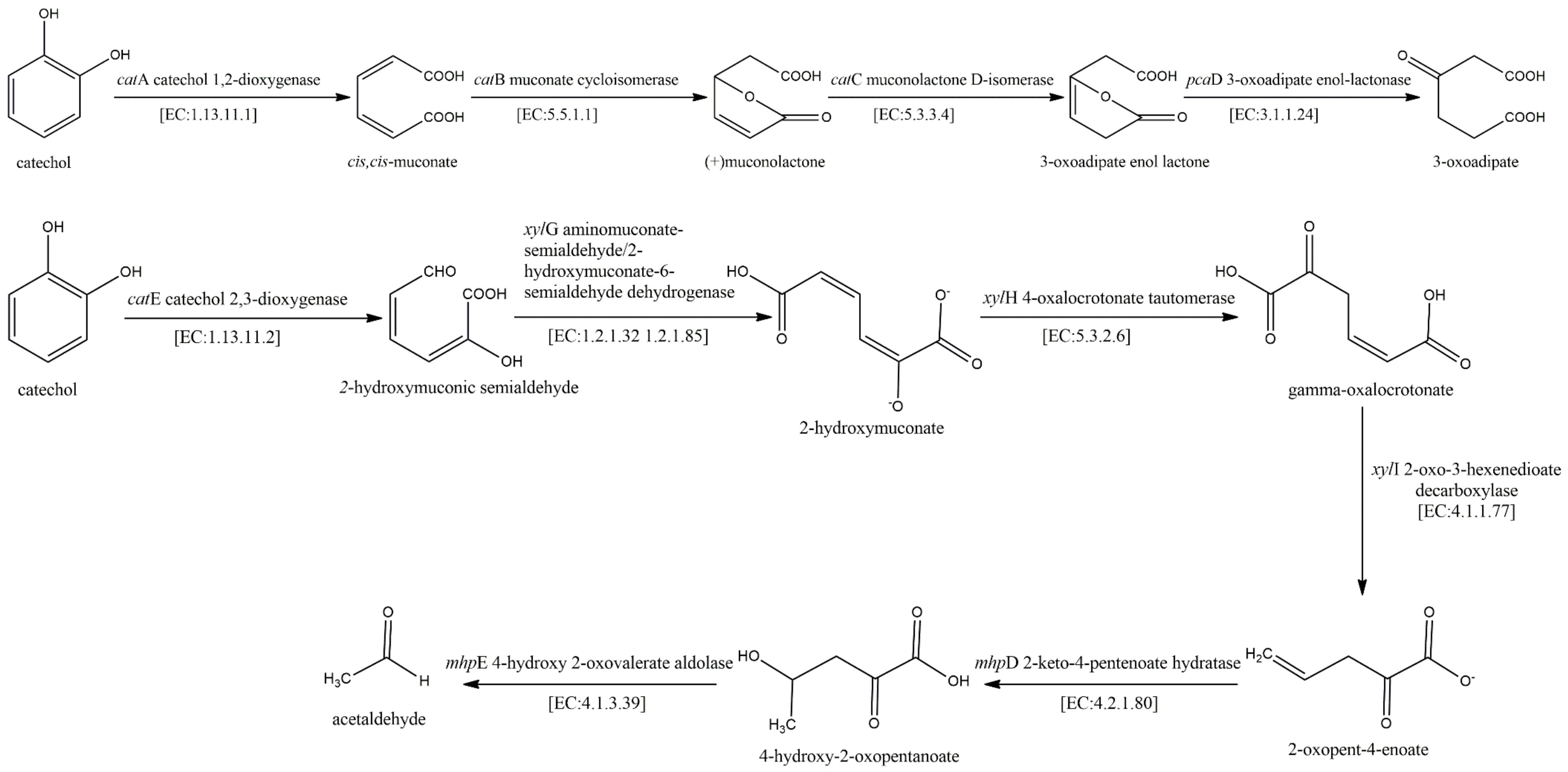

Figure S2. Schemes of (a) ortho- and (b) meta-pathways of catechol degradation

Table S2. Distribution of catechol dioxygenase genes in the genomes of representatives of different *Gordonia* species. Species where C2,3DO and C1,2DO are present simultaneously are marked green. Species where only C1,2DO is found are marked yellow. *Gordonia malaquae* is the only species whose strains contain only C2,3DO but no C1,2DO.

Number	*Gordonia* species	C23DO	C12DO
1	*aichiensis*	-	+
2	*alkanivorans*	-	+
3	*amarae*	+	+
4	*amicalis*	-	+
5	*asplenii*	+	+
6	*bronchialis*	+	+
7	*desulfuricans*	-	+
8	*hankookensis*	-	+
9	*hongkongensis*	-	+
10	*humi*	-	+
11	*hydrophobica*	-	+
12	*insulae*	-	+
13	*jacobaea*	-	+
14	*jinghuaiqii*	-	+
15	*lacunae*	+	+
16	*malaquae*	+	-
17	*mangrovi*	-	+
18	*namibiensis*	-	+
19	*neofelifaciens*	-	+
20	*oryzae*	+	+
21	*otitidis*	-	+
22	*paraffinivorans*	-	+
23	*phthalatica*	+	+
24	*polyisoprenivorans*	+	+
25	*pseudoamarae*	-	+
26	*rizosphera*	-	+
27	*rubripertincta*	+	+
28	*sihwensis*	-	+
29	*sputi*	-	+
30	*terrae*	+	+
31	*westfalica*	+	+
32	*zhaorongruii*	-	+
33	*zhenghanii*	-	+
